# Long Non-coding RNAs: Major Regulators of Cell Stress in Cancer

**DOI:** 10.3389/fonc.2020.00285

**Published:** 2020-03-20

**Authors:** Patrick Connerty, Richard B. Lock, Charles E. de Bock

**Affiliations:** Children's Cancer Institute, School of Women's and Children's Health, Lowy Cancer Centre, University of New South Wales (UNSW), Sydney, NSW, Australia

**Keywords:** lncRNA, metabolism, cell stress, oxidative stress, cancer, tumor suppressor gene, genotoxic stress

## Abstract

Cellular stress can occur in many forms; oxidative stress caused by reactive oxygen species (ROS), metabolic stress from increased metabolic programs and genotoxic stress in the form of DNA damage and disrepair. In most instances, these different types of cell stress initiate programmed cell death. However, in cancer, cells are able to resist cellular stress and by-pass growth limiting checkpoints. Recent findings have now revealed that the large and heterogenous RNA species known as long non-coding RNAs (lncRNAs) are major players in regulating and overcoming cancer cell stress. lncRNAs constitute a significant fraction of the genes differentially expressed in response to cell stress and contribute to the management of downstream cellular processes, including the regulation of key stress responses such as metabolic stress, oxidative stress and genotoxic stress. This review highlights the complex regulatory role of lncRNAs in the cell stress response of cancer by providing an overview of key examples from recent literature.

## Introduction

When the first draft of the Human Genome Project was completed in 2001 it came as a major surprise that protein-coding genes accounted for as little as 1–2% of the human genome (1). Initial thoughts were that this non-coding element of our DNA was merely genetic noise, left over DNA with no function or role and was therefore colloquially named “junk DNA.” However, it is now appreciated that most of the genome, while non-protein coding, is transcribed into RNA and these transcripts appear to be functionally different RNA species (2).

It has since been identified that our genome encodes for both long (>200 nucleotide) and short (<200 nucleotide) non-coding RNA species. Short-RNA species include microRNAs, short interfering RNAs, Piwi-interacting, and small nucleolar RNAs. All of these have distinct roles in either positively or negatively regulating gene expression via epigenetic and post-transcriptional regulation of target mRNAs (3). Long non-coding RNAs (lncRNAs), loosely described as non-protein-coding transcripts of >200 nucleotides, are a diverse group of RNA molecules which have been discovered to promote and inhibit gene expression via a variety of mechanisms (4).

Recently, considerable research has now shown that lncRNAs are important regulators of the cellular stress response and thereby implicated in the maintenance of human cancer. The aim of this review is to highlight some of the recent key developments for the role of lncRNAs in cellular stress in cancer.

## Long Non-Coding RNAs

lncRNAs are broadly defined as non-protein coding transcripts longer than 200 nucleotides. Similar to protein coding mRNA, the majority of lncRNAs are transcribed by RNA polymerase II, with some exceptions transcribed by RNA Pol III (5). These lncRNAs can also be poly-adenylated, spliced, expressed stably and localized in the nucleus, cytosol or mitochondria (6).

## Classification

**Figure 1:**
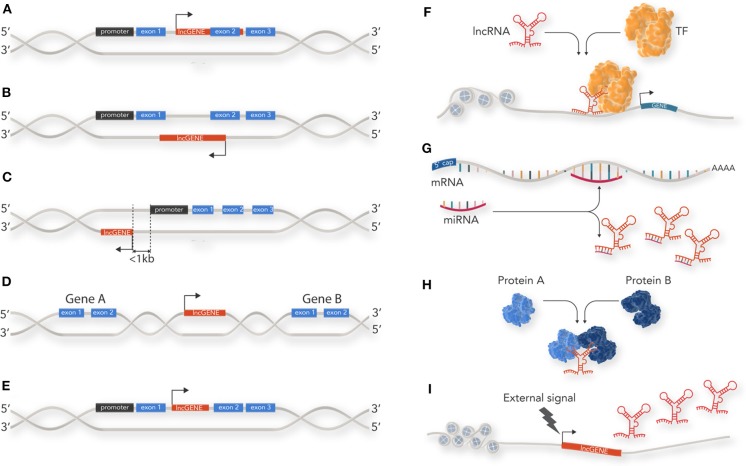
Classification and cellular function of lncRNAs: lncRNAs are classified by their location relative to coding loci along the genome. **(A)** sense lncRNAs are transcribed from the sense strand of protein-coding genes and contain exons from protein coding genes. **(B)** Antisense lncRNAs are the opposite of sense lncRNAs and are transcribed from the anti-sense strand of protein coding genes. **(C)** Bi-directional lncRNAs are similar to anti-sense lncRNAs but located in close proximity (within 1 kb) to the transcriptional start site of a protein coding gene and do not overlap, or only partially overlap, with their paired protein coding gene. **(D)** Intergenic lncRNAs (lincRNAs) do not intersect with any protein-coding gene annotations and are located in the long stretches of intergenic space present in the human genome. **(E)** Intronic lncRNAs are restricted to protein coding gene introns. **(F)** lncRNAs can act as guides for specific proteins and facilitate their localization within the cell or to a specific genetic locus to enable their action. For example, recruitment of transcription factor (TF) to a gene promotor. **(G)** lncRNAs can act as decoys by sequestering inhibitory RNAs, such as miRNAs, and prevent mRNA degradation. **(H)** lncRNAs can act as scaffolds for proteins within a larger protein complex. **(I)** lncRNAs can act as a cellular signal by which lncRNA transcription occurs at a very specific time and cellular location related to developmental cues or stimuli response.

There is wide literature on the classification of non-coding RNAs which is constantly being updated as new discoveries are made about this extensive RNA species. lncRNAs can be classified by a diverse range of features such as genomic location and biogenesis, lncRNA structure, protein binding motif (k-mers) and mechanism of action (7, 8). At their simplest level, however, lncRNAs can be classified by their location relative to coding loci along the genome into 5 main categories: (1) sense; (2) antisense; (3) bi-directional; (4) intergenic; and, (5) intronic (Figures 1A–E). Sense lncRNA are transcribed from the sense strand of protein-coding genes and contain exons from protein coding genes. They sometimes overlap with part of the protein-coding gene or cover the entire sequence of a protein coding gene through an intron. Anti-sense lncRNAs, as their name suggests, are the opposite of sense lncRNAs and are transcribed from the anti-sense strand of protein coding genes. Bi-directional lncRNAs are similar to anti-sense lncRNAs but located in close proximity (within 1 kb) to the transcriptional start site of a protein coding gene and do not overlap, or only partially overlap, with their paired protein coding gene. Intergenic lncRNAs (lincRNAs) do not intersect with any protein-coding gene annotations and are located in the long stretches of intergenic space present in the human genome. Finally, intronic lncRNAs are restricted to protein coding gene introns and are either independent unique transcripts or created as a by-product of the pre-mRNA splicing.

## Function

lncRNAs have diverse roles in cellular process such as acting as epigenetic modulators, promoting or inhibiting transcription, splicing, translation, and modulating protein function. lncRNAs achieve this through four main mechanisms (Figures 1F–I):

Guidance: lncRNAs can act as guides for specific proteins and facilitate their localization within the cell or to a specific genetic locus to enable their action. For example, one of the first lncRNAs documented, Xist, is able to accumulate on the entire length of the X chromosome and induce heterochromatin formation via recruiting components of the PRC2 polycomb protein complex. This results in X chromosome inactivation in humans and mice (9).

Decoy: lncRNAs can bind to and inhibit a protein target or titrate out protein or other non-coding RNA (such as a miRNA). Examples of this include the lncRNA GAS5 which contains hairpin structures which function as glucocorticoid receptor mimics, sequestering activated glucocorticoid receptor (10), or lncRNA-PAGBC which competitively binds miRNA to alleviate mRNA repression (11).

Scaffold: lncRNAs that serve as a central platform to recruit multiple proteins into ribonucleoprotein complexes. For example, HOTAIR, which acts as modular scaffold of histone modification complexes (12).

Signal: lncRNAs which may not necessarily have a direct biological function but can act as a cellular signal because their transcription occurs at a very specific time and cellular location—related to developmental cues or stimuli response. For example the lncRNA linc-p21 acts as a transcriptional repressor in the *p53* pathway and is upregulated directly in response to DNA damage (13).

It should be noted that these functions are not mutually exclusive, and some lncRNAs such as HOTAIR, have been documented to regulate gene expression through a combination of the above functional mechanisms. By facilitating the binding of histone modification complexes, HOTAIR acts as a modular scaffold, and by targeting the PRC2 complex to specific genomic locations, it serves as a guide. Furthermore, lncRNAs utilize the mechanisms of action outlined above to function as lncRNA oncogenes or lncRNA tumor suppressors in a cancer specific context. These two functional groups can be defined as a gene that encodes a lncRNA with the ability to directly promote tumorigenesis or a gene that encodes a lncRNA with the ability to directly inhibit tumorigenesis respectively. As our knowledge of lncRNAs role in oncogenic gene regulation widens so too does our understanding on the key roles these RNA molecules play in a cancer cells ability to survive cell stresses.

## The Cancer Specific Role of Long Non-Coding RNAs in Cellular Stress

In order for a cell to develop into a cancer it must overcome a number of anti-oncogenic checkpoints originally referred to as the “hallmarks” of cancer. Historically there were six hallmarks needed to be overcome for a cell to develop into a cancer. This list included; a cell to develop insensitivity to anti-growth signals, sustained angiogenesis, limitless replication potential, self-sufficiency in growth signals, and evasion of apoptosis (14). Recently, the list was updated to include immune system evasion and importantly, deregulation of metabolism (15) a central process in cellular stress responses.

A key feature of cancer cells is their ability overcome environmental stresses including but not limited to hypoxia, nutrient deprivation, and exposure to DNA-damaging agents (16). To survive these stressful conditions, cancer cells utilize the hallmarks of cancer and alter gene expression, reprogram metabolic pathways and evade growth inhibition signaling. lncRNAs have now been implicated in both positively and negatively regulating, metabolic stress, oxidative stress and genotoxic stress of cancer cells (Table 1) as well as an appreciable number of cancer-related cell signaling pathways (35).

**Table 1:** List of lncRNAs implicated in cancer cells in response to different stress types.

**lncRNA**	**lncRNA alias**	**Cancer**	**Stress type**	**Role**	**Reference**
MACC1-AS1	MACC1-AS1	Gastric	Metabolic	Pro-Oncogenic	(17)
GLCC1	AF339830	Colorectal	Metabolic	Pro-Oncogenic	(18)
SAMMSON	SAMMSON	Melanoma	Metabolic	Pro-Oncogenic	(19)
FILNC1	FILNC1	Renal	Metabolic	Anti-Oncogenic	(20)
IDH1-AS1	IDH-AS1	Colon/Cervical	Metabolic	Anti-Oncogenic	(21)
NBR2	NBR2	Breast	Metabolic	Anti-Oncogenic	(22)
HAND2-AS1	HAND2-AS1	Osteosarcoma	Metabolic	Anti-Oncogenic	(23)
H19	H19	Cholangiocarcinoma/ Pituitary	Metabolic/Oxidative	Both Pro and Anti Oncogenic	(24, 25)
HULC	HULC	Cholangiocarcinoma	Oxidative	Pro-Oncogenic	(25)
NLUCAT1	Lnc-ARRDC3-1	Lung adenocarcinoma	Oxidative	Pro-Oncogenic	(26)
NONHSAT1010169	NOAT113026	Breast	Genotoxic	Pro-Oncogenic	(27)
GUARDIN	LNCTAM34A	Breast	Genotoxic	Pro-Oncogenic	(28)
NEAT1	NEAT1	Multiple Myeloma	Genoxtoxic	Pro-Oncogenic	(29)
BORG	BMP/OP-responsive gene	Breast	Genotoxic	Pro-Oncogenic	(30)
PRAL	PRAL	Hepatocellular Carcinoma	Genotoxic	Anti-Oncogenic	(31)
LOC572558	HSALNT0149810	Bladder	Genotoxic	Anti-Oncogenic	(32)
LincRNA-p21	TP53COR1	Lung/Sarcoma/Lymphoma	Genotoxic	Anti-Oncogenic	(13, 33)
PANDA	PANDAR	Bone	Genotoxic	Anti-Oncogenic	(34)

## Metabolic Stress

**Figure 2:**
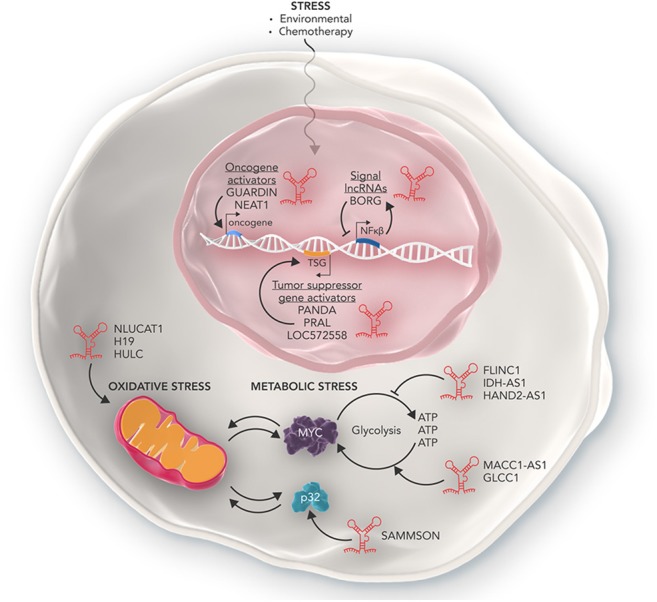
The many roles of lncRNAs in regulating cellular stress. Some lncRNAs such as NLUCAT1, H19, and HULC can promote the ability of cancer cells to overcome oxidative stress in the mitochondria. Other lncRNAs such as MACC1-AS1, GLCC1, and SAMMSON promote the ability of cancer cells to deal with increased metabolic stress and enables cancer cells to produce high amounts of metabolic energy required to rapidly proliferate. Conversely, lncRNAs such as FLINC1, IDH-AS1, and HAND2-AS1 act in an anti-oncogenic manner and inhibit the ability of cancer cells to tolerate metabolic stress via mechanisms such as inhibiting transcription of oncogenic metabolic proteins such as MYC. Furthermore, lncRNAs can also regulate the response of cancer cells to genotoxic stress as a result of chemotherapy. Some lncRNAs can activate oncogenes conferring resistance to genotoxic stress, while others activate tumor suppressor genes and inhibit cancer cell development. Other lncRNAs such as BORG are part of complex feedback loops which help maintain homeostasis of cancer cells in response to genotoxic stress.

The reprogramming of metabolism is now recognized as one of the hallmarks of cancer. Most cancers utilize an inefficient aerobic glycolysis pathway in favor of oxidative phosphorylation for their energy production (termed the Warburg effect) (36). This abnormal metabolic program allows cancer cells to produce the high levels of cellular energy needed for rapid proliferation. However, inefficient metabolic processing can also lead to an increase in stress as the cancer cells attempt to overwork metabolic networks. In order to deal with this increased stress burden cancer cells often over express key enzymatic proteins of cellular energy production pathways (such as AMPK, PKM2, MYC) or downregulate metabolic suppressors (such as p53). It is now becoming apparent that lncRNAs are key players in enabling cancer cells to reprogram metabolism and deal with metabolic stress (Figure 2) (37).

Some lncRNAs are able to act as oncogenes and enable cells to overcome metabolic stress loads through promoting the expression of key genes that facilitate metabolic plasticity. In gastric cancer cells the lncRNA MACC1-AS1, is able to stabilize its sense MACC1 RNA and post-transcriptionally increase MACC1 expression. MACC1 upregulation then mediates metabolic plasticity through the AMPL/Lin28 pathway and maintains the expression of key metabolic enzymes GLUT1, HK2, and LDH throughout glucose deprivation (17). Similarly, in colorectal cancer cells, the lncRNA GLCC1 is expressed in response to glucose starvation. GLCC1 then stabilizes the oncogenic transcription factor C-MYC from ubiquitination mediated degradation by directly binding with HSP90. This in turn allows the cells to survive high rates of glycolysis and lactate generation (18). Finally, in melanoma, the lncRNA SAMMSON was found to promote mitochondrial stability via sequestering p32, a key regulator of mitochondrial homeostasis and metabolism, in the cytoplasm where it stabilized mitochondria and promoted proliferation. Depletion of SAMMSON directly resulted in structurally aberrant mitochondria which were sensitive to accumulation of mitochondrial peptide precursors and mitochondrial import defects, collectively known as mitochondrial precursor-over-accumulation stress (mPOS) (19).

In contrast, other lncRNAs can act as tumor supressors to inhibit tumor cell survival and are often down regulated in cancer cells. In renal cancer cells, the lncRNA FILNC1 is significantly down regulated compared to healthy kidney cells. FILNC1 can directly bind with AUF1, a C-MYC mRNA binding protein and inhibits C-MYC mRNA processing resulting in the downregulation of C-MYC protein and altered metabolic plasticity (20). Similarly, the lncRNA IDH1-AS1 is transcriptionally repressed by C-MYC to promote the Warburg effect via HIF1α. Overexpression of IDH1-AS1 in colon and cervical cancer cells resulted in decreased cell proliferation and cancer xenograft growth. IDH1-AS1 was found to promote homodimerization of IDH1, a key protein of the TCA cycle, and decreased glycolysis (21). In breast cancer cells, the lncRNA NBR2 was found to be directly induced by the LKB1-AMPK pathway under energy stress. It in turn regulates AMPK, a critical sensor of cellular energy status, and promotes its kinase activity thus keeping the metabolic pathways of the cell in check. Depletion of NBR2 increased cell proliferation, decreased apoptosis and allowed breast cancer cells to continue cycling under high levels of energy stress (22). Finally, in osteosarcoma, the lncRNA HAND2-AS1 inhibited glucose uptake, lactate production and expression of metabolic enzymes via sequestering FBP1, an inhibitory enzyme of the metabolic gene HIF1α. Furthermore, expression of HAND2-AS1 was directly increased after metabolic stress within the cell. RNAi induced knockdown of HAND2-AS1 relieved the metabolic energy stress induced apoptosis, resulting in cancer cell survival and promoted osteosarcoma progression (23).

However, it should be noted that lncRNAs can act as both tumor suppressors and oncogenes in regulating the cell stress response. For example, whilst overexpression of the lncRNA H19 promotes cholangiocarcinoma cell growth under oxidative stress conditions, in pituitary tumors H19 has been found to act as a tumor suppressor lncRNA and inhibits the ability of cells to respond to metabolic stress. In the pituitary tumor context, H19 directly binds with 4E-BP1 and competitively inhibits it binding to the key energy sensing protein mTORC1, resulting in reduced 4E-BP1 signaling. When H19 is downregulated, 4E-BP1 is able to re-bind mTOR subunits, increasing phosphorylation to prevent its interaction with translation factor elF4E, resulting in increased protein translation, metabolic flexibility, and tumorigenesis. Significantly, over expression of H19 was more effective than the dopamine agonist cabergoline, the first-line treatment for pituitary tumors, at inhibiting tumor cell growth in *in vivo* models (24). Taken together, this highlights that importance of cellular context for lncRNA function and has implications on targeting lncRNAs as part of any future therapeutic strategy.

## Oxidative Stress

Reactive oxygen species (ROS), are the natural by-products of aerobic metabolism in the cell. Mitochondria are the primary source of endogenous ROS. These metabolic molecules play an important role in the physiological function of cells, as both effectors and as signaling molecules. Due to their central role and their potential toxic impact on cellular components, ROS production and removal are tightly controlled processes (38). Cancer cells are often present in hypoxic microenvironments that promote an accelerated metabolism that demands high ROS concentrations for increased proliferation. Different mechanisms are employed by cancer cells to facilitate the high levels of oxidative stress, such as utilization of the pentose phosphate pathway and deregulation of key antioxdative proteins such as NRF2 (39). However, two recent studies have highlighted roles for lncRNAs in the ability of cancer cells to respond to oxidative stress. In cholangiocarcinoma the lncRNAs H19 and HULC are upregulated in cells as a direct response to exposure to oxidative stress factors such as hydrogen peroxide. These then in turn act as miRNA sponges and sequester let-7a, let-7b and miR-372/miR-373 thereby up-regulating the expression of the cytokine IL-6 that promotes migration and cell invasion in *in vitro* assays (25). In a second study on lung adenocarcinoma, lncRNA NLUCAT1 expression directly promoted the expression of key oxidative homeostasis genes *ALDH3A1, GPX2, GLRX*, and *PDK4* and therefore the ability of lung cancer cells to resist ROS-induced apoptosis. Consequently depletion of NLUCAT1 could re-sensitize cells to ROS-dependent apoptosis induced by hydrogen peroxide (26).

## Genotoxic Stress

Cells undergo genotoxic stress as a result of damage to DNA structure and genome instability (40). The cellular mechanisms of DNA-damage prevention, such as DNA repair, cell cycle checkpoints and apoptosis, all protect cells from acquiring deleterious genetic mutations. However, genotoxic stress often induces carcinogenesis through dysregulating key regulatory pathways of the cell (41). Remarkably in fully established cancer cells, the properties of genotoxic stress are used to treat the disease. The basis of many chemotherapeutic agents is to induce DNA damage and use genotoxic stress responses to induce cell death in cancer cells. However, in therapy-resistant cases, cancer cells can adapt to resist, and overcome genotoxic stress. Cancer cells can resist treatment and genotoxic stress through a variety of mechanisms such as inhibiting tumor suppressor genes, up regulating cellular growth factors and skipping cell cycle checkpoints (42). lncRNAs also play a key role in the ability of cancer cells to overcome genotoxic stress and directly contribute to carcinogenesis, therapy resistance and aggressiveness of cancers (Figure 2).

As the fundamental basis of many current chemotherapeutic therapies is to induce genotoxic stress to kill cancer cells, drug resistance and acquired chemoresistance have been identified to be closely associated with genotoxic stress resistance. The altered expression of lncRNAs has now also been identified as an important mechanism to directly promote drug resistance in cancer cells (43). For example, over expression of the lncRNA NONHSAT1010169 in breast cancer tumors, induced resistance to the anthracycline epirubicin, a first-line treatment for metastatic breast cancer. Moreover, forced expression of NONHSAT101069 stimulated the migration and invasion of breast cancer cells, while its depletion re-sensitized resistant breast cancer cells to epirubicin. At the molecular level, it was found that NONHSAT101069 drove epirubicin resistance via sequestering miR-129 which in turn relieved miRNA-inhibition of the oncogenic protein Twist1, increasing drug resistance (27). Another newly described and annotated lncRNA in breast cancer is GUARDIN, a p53-responsive lncRNA, that can sustain cancer cell growth via two key mechanisms; sequestering miR-23a which in turn stabilizes *TRF2* and acting as an RNA scaffold for the oncoprotein BRCA1. Through these interactions GUARDIN is able to protect cells from apoptosis induced by genotoxic stress and drive cancer cell resistance to chemotherapies (28). In multiple myeloma cells, the lncRNA NEAT1 upregulates DNA-repair proteins and enables multiple myeloma cells to resist massive amounts of genotoxic stress. As anticipated, knockdown of NEAT1 results in a downregulation of DNA-repair processes and re-sensitizes multiple myeloma cells to common chemotherapeutic agents (29).

In other cases, lncRNAs are able to be directly induced by genotoxic stress and provide resistance to cancer cells as a type of pro-cancer cell-stress response. One example is the lncRNA BORG. BORG is induced within breast cancer cells which have been exposed to environmental and chemotherapeutic stresses commonly faced by cancer cells undergoing treatment. Exposure of breast cancer cells to doxorubicin results in a rapid and marked increase in the expression of BORG. This expression is driven by NF-kB, which in a feed-forward loop manner drives NF-kB activity and provides breast cancer cells with chemoresistance properties reducing the extent of DNA damage caused by chemotherapeutic agents. Knockdown of BORG results in re-sensitization of breast cancer cells. BORG provides a great example of how the synthesis of lncRNAs is extremely rapid, compared to protein synthesis, and thus how lncRNAs are ideal genetic tools that can be readily deployed when cells are subject to stress (30).

Alternatively, lncRNAs can also inhibit the ability of cancer cells to respond to genotoxic stress. This is often through enhancing DNA-damage response pathways and promoting genotoxic induced cell cycle arrest and apoptosis. One of the most well-studied and iconic enzymes of the DNA-damage response pathway is the tumor suppressor gene *TP53*. The p53 protein regulates the expression of hundreds of genes that are involved in multiple biological processes, including DNA damage repair, cell cycle arrest and apoptosis. In the case of genotoxic stress, p53 is considered the key “decision making” transcription factor that determines cellular outcomes (44). lncRNAs are now found to be positively promoted or associated with p53 and the DNA-damage pathway. PRAL is one such p53 associated lncRNA. PRAL is a hepatocellular carcinoma related lncRNA whose genomic alterations are significantly associated with hepatocellular carcinoma patient survival. PRAL can inhibit the growth of hepatocellular carcinoma and induce apoptosis via p53 in both *in vitro* and *in vivo* settings. It achieves this by facilitating the combination of HSP90 and p53 through RNA-scaffolding and thereby inhibiting p53 ubiquitination and subsequent degradation (31). Another example of p53 enhancing lncRNAs is LOC572558. Over expression of the lncRNA LOC572558, which is found to be down regulated in bladder cancer, is able to enhance the phosphorylation of p53. This in turn enhances p53 signaling to inhibit bladder cancer cell proliferation (32). Furthermore, the LincRNA-p21 is a vital enabler of p53. LincRNA-p21 is essential to p53-mediated apoptosis in response to DNA damage. It does this by recruiting hnRNP-K to increase p53-dependent transcription of p21 (a key checkpoint protein in the p53 pathway). After lincRNA-p21 depletion, hnRNP-K binds to the promoters of p53-repressed genes, this results in increased proliferation rates and faulty G1/S check points (33, 45). Many lncRNAs have documented supportive effects on p53 activity and stability, however, the exact mechanisms by which this is achieved remains unknown. For example, the lncRNA PANDA stabilizes p53 protein, not mRNA, expression and protects it from proteasome degradation via an as yet, unknown mechanism (34). The role of lncRNAs in regulating the p53 signaling network is such an extensive and emerging field in the space of lncRNA biology that a dedicated database TP53LNC-DB has been recently created which annotates currently available information of lncRNAs in human p53 signaling (46).

Together this highlights the ability of lncRNA in cancer cells to resist genotoxic stress, their ever-growing value as biomarkers for diagnosing cancer, and as therapeutic targets for developing new treatments for drug resistant cancers or inhibiting cancer cell progression (47, 48).

## Concluding Remarks

In conclusion, the ability of cancer cells to survive and react to cellular stresses is one of the key features which defines their capacity to cause disease and evade therapy. We are now appreciating that there is a secondary “non-coding” level of complexity which governs cancer stress responses to a range of diverse pressures. The key RNA species, lncRNAs, are able to modulate genetic regulation and cellular pathways in an oncogenic manner allowing cancer cells to survive high levels of cellular stress that would otherwise kill healthy cells. Importantly, lncRNAs are also able to act as tumor suppressors in regulating cellular stress, with the same lncRNA performing opposite roles in different tissues, highlighting the tissue-specific complexity of these RNA molecules. While this review is far from an exhaustive list of lncRNAs in regulating cancer cell stress, the studies highlighted in this review exemplify the pleiotropic effects of lncRNAs on cell stress in cancer. Future studies will continue to elucidate more functions of lncRNAs in cancer-stress responses that will help provide important insight into lncRNA action, allowing us to harness them for therapeutic strategies and proving that this “junk” isn't junk at all.

## Author Contributions

PC, RL, and CB all made substantial, direct and intellectual contributions to this manuscript, and approved it for publication.

### Conflict of Interest

The authors declare that the research was conducted in the absence of any commercial or financial relationships that could be construed as a potential conflict of interest.
